# A research-driven flowchart to approach change in couples

**DOI:** 10.3389/fpsyg.2024.1438394

**Published:** 2025-01-06

**Authors:** Francesca Capozzi

**Affiliations:** CORE | Cognitions and Relations Laboratory, Social Psychology and Personality Section, Department of Psychology, UQAM | Université du Québec à Montréal, Montreal, QC, Canada

**Keywords:** common factors, couple therapy assessment, couple therapy goals, relational therapy, relationship science

## Abstract

In most cases, couple therapists systemically see couples’ distress as the result of reciprocal maladaptive patterns to which each partner contributes. Yet, therapists can struggle to share with the couple such a relational understanding of their distress and identify goals for change accordingly, as structured, step-by-step methods are not available in the extant literature. This perspective paper reviews research across various domains of relationship science to summarize cohesively the best practices for goal identification in a step-by-step flowchart. The flowchart is divided into three main areas, derived from the available literature: establishing couple therapy appropriateness, determining general goals, and conceptualizing specific goals from a systemic perspective. Aimed at facilitating training, the resulting recommendations will broadly support a goal-focused approach to systemic assessment.

## 1 Introduction

Couple therapy is systemic in nature. Except for intimate partner violence, most couples go to therapy dancing the “tango” of reciprocal patterns that take the form of so-called bow-tie patterns or self-perpetuating loops (Furrow et al., 2022), in which Partner A’s behavior triggers or confirms Partner B’s emotional insecurity or cognitive distortions eliciting behavior that triggers or confirms Partner A’s emotional insecurity or cognitive distortion.

Consider the example of Aiden and Alex. Aiden is cold and absent and often works late or spends the evenings after dinner in their office to have some peaceful time. Alex is critical and resentful and often feels alone and overwhelmed by the responsibility of keeping the house clean and the relationship afloat. When they come to therapy, they lament a strained connection, each portrays the other’s behavior as the problem, and no hope seems possible. However, the therapist sees that the behavior of one pushes the other further away and vice versa and that change must occur on both sides to be effective. How can the therapist share this perspective with the couple and help them identify goals accordingly?

The saying the relationship is the client—often so obscure to trainees—precisely refers to the key systemic approach that sees couples’ distress as the result of reciprocal chains of behavior/interpretation/responses to which each partner contributes. Yet, despite its importance, structured guidance to build this relational understanding seems missing in the available literature. For example, common factor theory addresses factors contributing to therapy success across different clinical approaches and has gained primary relevance in research and training (Davis and Hsieh, 2019). Applied to couple therapy, the theory emphasizes relational conceptualization as a key common principle and identifies the disruption of dysfunctional relational patterns as a key systemic goal (Davis et al., 2012; Bradbury and Bodenmann, 2020).

The advantage of common factor approaches is that their indications can apply to virtually all therapeutic approaches, laying a strong theoretical foundation for understanding therapeutic success across models (Fife et al., 2014). As such, the present perspective falls firmly within this tradition. However, these approaches often lack concrete, step-by-step indications on how to align systemic conceptualizations with specific therapy goals (see for example Chambers, 2012; Stanton and Welsh, 2012). Additionally, these approaches often lack connection with other areas of relationship science emphasizing key elements of therapeutic success, like the importance of evaluating couple therapy appropriateness, formulating a distinction between general and specific goals, and identifying cognitive and affective factors that promote change (Bradbury and Bodenmann, 2020).

The present perspective addresses these issues by organizing recommendations on identifying couples’ goals for change in a step-by-step flowchart. Integrating literature from various domains of relationship science, the proposed flowchart provides a practical tool to standardize goal identification from a systemic perspective encompassing general, specific, and individual goals for change. Aimed at facilitating training, the resulting recommendations will broadly support a goal-focused approach to systemic assessment.

## 2 Change in couples

Effective change starts with a shared and client-focused understanding of the couple’s needs. However, couples often enter therapy without a relational understanding of their issues (Benson et al., 2012), and self or other blaming are more common views, especially in individualistic societies (Stanton and Welsh, 2012). Thus, it can be challenging for the therapist to guide clients toward a relational understanding of their problems. Going back to the example of Aiden and Alex, the therapist may struggle to show Aiden that their behavior communicates a lack of interest and to show Alex that their behavior communicates contempt. Additionally, Aiden and Alex may be hesitant to take responsibility for their contribution to the relational distress, which may represent yet another challenge for the therapist in the absence of structured methods to translate systemic conceptualizations into actionable goals that resonate with the client’s perspective (Sperry, 2005; Eldridge et al., 2022).

**Figure 1:**
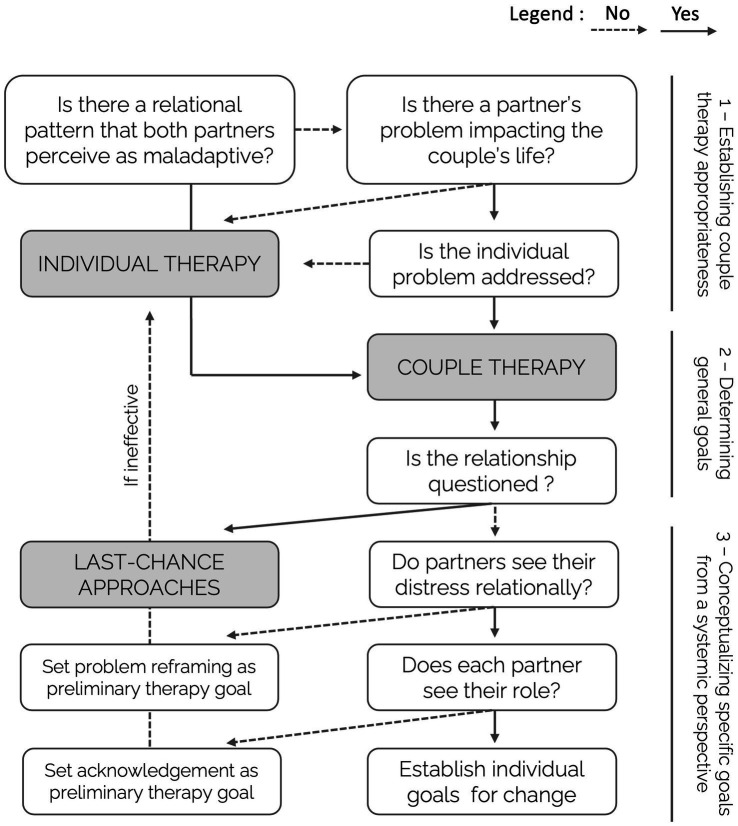
Flowchart recommendations to identify couples’ goals for change from a systemic perspective. Solid lines represent yes-responses; dashed lines represent no-responses. Couple therapy is appropriate when there is a maladaptive relational pattern or when an individual partner’s problem impacts the couple’s life, but such individual problem is addressed otherwise. If the couple is questioning the continuation of the relationship, last-chance approaches should be preferred. Finally, therapy should focus on reframing the problem relationally if the partners struggle to take this perspective and on responsibility acknowledgment or individual goals for change according to their ability to see their individual contribution to the relational pattern.

What follows reviews research across various domains of relationship science to summarize cohesively the best practices for goal identification in a step-by-step flowchart (Figure 1). The flowchart is divided into three main areas, derived from the available literature and described below: establishing couple therapy appropriateness, determining general goals, and conceptualizing specific goals from a systemic perspective.

### 2.1 Establishing couple therapy appropriateness

As paradoxical as it may seem, research suggests that determining the appropriateness of couple therapy may be the first step of an ideal flowchart identifying couples’ goals for change (Figure 1, 1). Couple therapy’s appropriateness extends beyond relational distress (Halford and Pepping, 2019). For example, couple therapy can be recommended when an individual partner’s issues significantly affect the couple’s functioning (e.g., Alder et al., 2018) and has historically proven effective in easing such issues from relational contributing factors (e.g., Wittenborn et al., 2022). For example, Aiden’s behavior may reflect depression or dysthymia, which does not exclude couple therapy insofar as their mood issues may affect and be affected by relational issues (Montesano et al., 2014).

However, therapists and approaches differ in how much they stress the importance of addressing individual issues with separate individual therapy. Classic systemic approaches would encourage systemic therapy as a primary treatment even in cases in which there is a so-called Identified Patient (IP) based on behavioral or psychiatric issues (Carr, 2019; Hogue et al., 2022; Wittenborn et al., 2022), whereas other approaches present less agreement (e.g., Isakson et al., 2006). For example, individual therapy is recommended for individual circumstances jeopardizing discernment capacity and behavioral control, such as substance misuse (Klostermann and Fals-Stewart, 2004) or anger and violence issues (Karakurt et al., 2016). Other issues have received less consensus, like various forms of relational trauma that impact and are impacted by the relational dimension. In these cases, viable approaches include providing a safe space to support partners’ reciprocal understanding and the navigation of trauma triggers; these important topics extend beyond the scope of this perspective, but interested readers can refer to the appropriate literature (e.g., MacIntosh and Johnson, 2008; MacIntosh, 2017; Kleiner-Paz and Nasim, 2021).

While acknowledging that the therapist’s case-by-case recommendations will largely depend on various factors, including the relational context and the specific individual issue, the present perspective encourages careful consideration before moving forward with couple therapy without recommending additional individual therapy when there is evidence that the maladaptive relational patterns can be largely attributed to the behavior of one partner. For example, Alex’s best intentions (and behaviors) may prove ineffective if Aiden’s mood instability depends on factors that extend beyond the relationship. Additionally, focusing on reciprocally triggering patterns of action, interpretation, and response may prove tricky in these circumstances. When a partner’s individual issues are addressed otherwise, maintaining the focus on the relational patterns is easier, and the risk of inappropriate alliances with the other partner is reduced (Gurman and Burton, 2014; Friedlander et al., 2018; Sotero and Relvas, 2021).

This is especially important when the imbalance is evident and jeopardizes safety, like with intimate partner violence (for various perspectives on this topic: Stith et al., 2012; Antunes-Alves and De Stefano, 2014; Karakurt et al., 2016; Hurless and Cottone, 2018). In these cases, individual therapy is strongly recommended, and couple or family approaches that support individual or subsystem recovery from psychological wounds may be preferable (e.g., Diamond et al., 2016).

In sum, couple therapy is the most appropriate when there is a relational pattern that is perceived as maladaptive and when an individual partner’s problem significantly impacts the couple’s life, but such individual problem is addressed with some form of individual therapy; otherwise, individual therapy should be encouraged (Figure 1, 1).

### 2.2 Determining general goals for relationship continuation

Another critical assessment concerns overall goals for therapy. Specifically, research recommends assessing whether the couple questions their commitment to the relationship (Halford et al., 2016) and tailor treatment accordingly (Owen et al., 2012; Doherty et al., 2016). Thus, the second step of the flowchart should be determining whether partners aim at relationship improvement or are rather questioning the relationship continuation (Figure 1, 2).

Couples considering dissolution include couples experiencing high conflict, substantial misalignments in life projects, significant betrayals in trust or safety, and/or seemingly irremediable loss of intimacy (Tremblay et al., 2008; Boisvert et al., 2011; Fraenkel, 2019). Interestingly, these areas substantially overlap with the areas identified in non-clinical, process-focused areas of relationship science to predict relationship quality, supporting the notion that perceived relationship quality is one of the key predictors of relational process and clinical outcomes (Ogolsky et al., 2017; Joel et al., 2020; Itzchakov et al., 2022; Righetti et al., 2022). Similarly, ambivalence toward the relationship has been shown to play a significant role in relational difficulties and the overall perception of relationship quality (Faure et al., 2022; Zoppolat et al., 2022, 2024).

Thus, clarifying commitment at the beginning of treatment is strongly recommended. If for example, Alex is questioning their ability or interest in continuing the relationship, there may be no sufficient safety or trust to expose Aiden’s longing for connection likely hiding behind their withdrawing behavior (Kula et al., 2024). In these cases, so-called last-chance approaches focusing on behavioral issues (Fraenkel, 2019) and/or an in-depth exploration of commitment and ambivalence (Owen et al., 2014) may be the most appropriate. Decoupling support may ultimately be considered if separation is decided (Lebow, 2019). To expand on these important topics, interested readers can refer to the relevant literature (e.g., Owen et al., 2014; Fraenkel, 2019; Lebow, 2019; Fishbane et al., 2020; Lebow and Snyder, 2022).

In sum, once couple therapy appropriateness has been established, general goals must be determined in terms of commitment to relationship continuation; if the relationship is questioned, last-chance approaches should be preferred (Figure 1, 2).

### 2.3 Conceptualizing specific goals from a systemic perspective

Arguably, however, most couples come to therapy with the general goal of improving the relationship, with relatively clear ideas of the life domains in which their distress is the most significant (Halford and Pepping, 2019). For example, research includes communication issues, insufficient (emotional) intimacy, and power unbalances among the main reasons for consultation (Doss et al., 2004; Roddy et al., 2019). Interestingly, these areas substantially overlap with the areas identified in process-focused areas of relationship science to predict relationship quality and duration, validating clients’ subjective experience of their presenting issues (Ogolsky et al., 2017; Joel et al., 2020; Itzchakov et al., 2022).

What couples often lack, however, is a relational understanding of their distress that rests on the acknowledgment of their interlocking behavioral patterns or feedback loops, focusing on reciprocal sustaining factors rather than individual behaviors, as described above. Critically, individual partners and couples may differ significantly in their willingness to frame the presenting problem relationally (Gurman and Burton, 2014). At this point of the chart, the therapist should assess the partners’ ability to see the relational nature of their presenting issue with minimal stimulation or psychoeducation (Figure 1, 3).

For example, Aiden and Alex’s situation can be seen as a typical pursue/withdraw pattern between a pursuing/expressive partner and a withdrawing/shunning partner stuck in a cycle of seemingly incompatible actions and responses (one pursues, and the other withdraws; Wile, 2013). Such strategies have been historically interpreted as individual differences in attachment strategies (Mikulincer and Shaver, 2019), although recent research indicates that they may also be a function of a couple’s specific patterns of interaction that emerged over time (Baucom et al., 2015; Brandão et al., 2020; Leo et al., 2021).

What ensures the maladaptive stability of this type of pattern is that partners reciprocally trigger each other’s insecurities (Mikulincer and Shaver, 2019): one (the pursuer) seeks emotional regulation via reassurance and connection and fears abandonment; the other (the withdrawer) seeks emotional regulation via autonomy and separation and fears enmeshment. Thus, when conflict-related distress calls for emotional regulation, each partner displays the behaviors that are most likely to confirm the partners’ relational fears in what can easily become an infinite loop of negative interactive cycles. Explained to clients in an accessible language and culturally compatible way, this view decreases maladaptive processes (such as blame, emotional reactivity, and isolation) and increases positive relational behaviors (such as compassion, empathy, and cooperation) (Fishbane and Wells, 2015; Fishbane, 2023).

This relational view of distress similarly applies to various presenting problems. In addition to the pursue/withdraw pattern, and as mentioned above, frequent reasons for consultation include communication issues, insufficient support, and unbalances in power (Doss et al., 2004, 2022; Roddy et al., 2019). Critically, research shows that communication issues contextually depend on bidirectional obstacles in both expression and listening (e.g., Gottman and Gottman, 2015; Righetti et al., 2022), support is connected to reciprocity in responsiveness (e.g., Itzchakov et al., 2022; Smoliak et al., 2022), and power imbalances are linked to reciprocal patterns of disempowerment and power misattributions (e.g., Knudson-Martin, 2013; Körner and Schütz, 2021). Thus, various areas of couple (dis-)functioning can be seen through the systemic lens of partners’ reciprocal influences.

In sum, once dissolution has been excluded and therapy improvement is set as a general goal for therapy, partners must be encouraged to frame their presenting problem in relational terms; when this is not possible, such framing should be set at the therapy focus (Figure 1, 3).

### 2.4 Pushing systemic understanding further

Corroborating a relational understanding, early solo and joint sessions should stimulate each partner’s reflections on their contribution to the relational pattern and establish directions for individual change. Partners’ willingness to take responsibility for their part in their relationship issues may be foundational to interrupting blaming cycles (Patrika and Tseliou, 2016; Smoliak et al., 2021) and implementing the therapist’s suggestions (Davis and Piercy, 2007). Additionally, focusing on individual change empowers the partners to contribute positively to change in their relationship, benefitting each partner’s sense of competence and autonomy (Halford, 2003; Knee et al., 2013) while also ensuring accountability (Fishbane, 2023). Thus, while this assessment does not have to be formal, it should be explicit (although formal tools can benefit the process; Halford and Pepping, 2019; Lavee and Avisar, 2006; Snyder et al., 2005).

Such understanding would ideally be framed as goals for individual change aiming to interrupt the current negative interaction cycle and drive the relationship toward the desired state. Thus, for example, Aiden’s sense of responsibility would benefit from the goal of reducing their impulse to pursue or criticism as it would empower them to change something without waiting for a change in Alex; similarly, Alex would feel empowered by the possibility of focusing on their ability to counteract their perceived passive role in the relationship.

Focusing on individual change may seem at odds with systemic approaches. However, multiple systemic approaches view self-regulation and agency as key elements of empathy, cooperation, and mutual understanding. For example, differentiation, defined as the ability to separate self from others, empowers individuals to make choices that are consistent with their personal and relational values while it legitimates and validates self and other experiences (Fishbane and Wells, 2015; McDowell et al., 2019; Knudson-Martin et al., 2021; see Rodríguez-González et al., 2020 for cross-cultural considerations). Quantitative research has highlighted the importance of perceived agency in fundamental relational processes such as accommodation (e.g., Kluwer et al., 2020), balancing personal and relational concerns (e.g., Visserman et al., 2017), and relational altruism (e.g., Gordon and Chen, 2013). Additionally, more differentiated individuals can better identify and regulate emotions (Kashdan et al., 2015), benefitting relational abilities within and outside the therapeutic setting (Tamir, 2016).

In sum, the final step of the proposed flowchart for couples’ assessment would be to determine each partner’s ability to understand the presenting problem as a relational pattern and acknowledge their contribution to such a pattern. When such individual goal setting is not possible, the therapy should reframe goals with such focus (Figure 1, 3).

## 3 Discussion

This perspective paper proposes a structured, research-driven approach to assess and promote change in couples from a systemic perspective encompassing general, specific, and individual goals for change. In the proposed view, systemic conceptualization is foundational to couple therapy, and partners’ acknowledgment of individual responsibility is a beneficial step to therapeutic change.

What if partners do not acknowledge their role in maintaining the status quo? As mentioned before, couples’ systemic understanding of their issues is often limited, and partners struggle to acknowledge their role in the relational pattern. In these cases, the present perspective encourages practitioners to consider negotiating such an understanding as the therapy goal. If such an understanding is ultimately not possible, therapy discontinuation and/or individual therapy could be considered. Reasons for such a stance include ethical and therapeutic considerations.

Ethically, the relational mandate of couple therapy must be clear to clients as they engage in couple therapy. As consent is a continuous process, reinforcing the relational therapeutic setting is arguably a therapeutic intervention in itself (Hecker and Murphy, 2018). Therapeutically, and as articulated above, a couple’s ability to understand their presenting issues from a relational perspective may be a key factor contributing to treatment success by reducing blame and increasing individual responsibility and agency, showing the foundational nature of systemic understanding in couple therapy (Becvar and Becvar, 2019).

Importantly, the proposed approach to goal identification is agnostic with respect to the method that is used to pursue the goals and to the clinical model that is privileged and can be applied in future research and practice from a multitude of perspectives (e.g., cognitive, emotional, socio-relational; Knudson-Martin et al., 2015). Additionally, there is no in-principle contra-indication to apply the proposed methods independent of clients’ cultural background, although client-focused cultural considerations are strongly encouraged in addressing the content of the goals (e.g., Rodríguez-González et al., 2020; Capozzi, 2022; Ealami and Hooshmandi, 2024; see also Trexler, 2024).

In conclusion, this paper provides a roadmap to frame couples’ distress systemically and identify general, specific, and individual goals for change accordingly. As such, this paper extends the current literature by suggesting a meta-theoretical approach to change in couples, which has the potential to benefit research, practice, and training in a wide range of therapy approaches. If Aiden and Alex decide to continue their relationship, their best chance will ultimately derive from their ability to change their views on their distress, acknowledge their individual responsibilities, and work jointly toward their new goals.

## Data Availability

The original contributions presented in the study are included in the article/supplementary material, further inquiries can be directed to the corresponding author.
